# Dysregulated mechanisms underlying Duchenne muscular dystrophy from co-expression network preservation analysis

**DOI:** 10.1186/s13104-015-1141-9

**Published:** 2015-05-03

**Authors:** Kavitha Mukund, Shankar Subramaniam

**Affiliations:** Bioinformatics and System Biology Graduate Program, University of California San Diego, 9500 Gilman Drive, MC0412, La Jolla, CA 92093 USA; Departments of Bioengineering, Computer Science & Engineering, Cellular & Molecular Medicine and Chemistry & Biochemistry University of California, San Diego, 9500 Gilman Drive, MC0412, La Jolla, CA 92093 USA

**Keywords:** Skeletal muscle, Duchenne muscular dystrophy, Co-expression network, Preservation statistics, Differential network analysis

## Abstract

**Background:**

Duchenne Muscular Dystrophy (DMD) is an X-linked recessive disorder with its primary insult on the skeletal muscle. Severe muscle wasting, chronic inflammation and fibrosis characterize dystrophic muscle. Here we identify dysregulated pathways in DMD utilizing a co-expression network approach as described in Weighted Gene Co-expression Network Analysis (WGCNA). Specifically, we utilize WGCNA’s “preservation” statistics to identify gene modules that exhibit a weak conservation of network topology within healthy and dystrophic networks. Preservation statistics rank modules based on their topological metrics such as node density, connectivity and separability between networks.

**Methods:**

Raw data for DMD was downloaded from Gene Expression Omnibus (GSE6011) and suitably preprocessed. Co-expression networks for each condition (healthy and dystrophic) were generated using the WGCNA library in R. Preservation of healthy network edges was evaluated with respect to dystrophic muscle and vice versa using WGCNA. Highly exclusive gene pairs for each of the low preserved modules within both networks were also determined using a specificity measure.

**Results:**

A total of 11 and 10 co-expressed modules were identified in the networks generated from 13 healthy and 23 dystrophic samples respectively. 5 out of the 11, and 4 out of the 10 modules were identified as exhibiting none-to-weak preservation. Functional enrichment analysis identified that these weakly preserved modules were highly relevant to the condition under study. For instance, weakly preserved dystrophic module D2 exhibited the highest fraction of genes exclusive to DMD. The highly specific gene pairs identified within these modules were enriched for genes activated in response to wounding and affect the extracellular matrix including several markers such as SPP1, MMP9 and ITGB2.

**Conclusion:**

The proposed approach allowed us to identify clusters of genes that are non-randomly associated with the disease. Furthermore, highly specific gene pairs pointed to interactions between known markers of disease and identification of putative markers likely associated with disease. The analysis also helped identify putative novel interactions associated with the progression of DMD.

**Electronic supplementary material:**

The online version of this article (doi:10.1186/s13104-015-1141-9) contains supplementary material, which is available to authorized users.

## Background

Duchenne muscular dystrophy (DMD), is a lethal form of dystrophinopathy characterized by marked deficiency or absence of subsarcolemmal cytoskeletal protein- dystrophin. Absence of this protein is caused due to frame shift mutations of the dystrophin gene [1]. Dystrophin, part of the dystroglycan complex plays a crucial role in maintaining the integrity of the muscle fiber. Absence of dystrophin causes uneven mechanical force transmissions leading to sarcolemmal ruptures and subsequent atrophy. Clinical manifestations of DMD occur by second year of birth and progressively degrade with time. The first decade of life is marked by developmental delays, and steady decreases in the strength of the limbs and torso with subsequent loss of ambulation. Respiratory and cardiac complications arise by the second decade of life leading to death [2]. Here we utilize a co-expression networks approach to gain insights into molecular interactions dysregulated in dystrophic skeletal muscle with respect to healthy muscle.

Co-expression networks are being increasingly used for deciphering disease mechanisms and providing systems level views of dysregulated pathways [3,4]. The basic premise of co-expression analysis is that strongly correlated genes are likely to be functionally associated. Weighted Gene Co-expression Network Analysis (WGCNA) is an open source tool that performs co-expression analysis using a network theoretic approach. WGCNA integrates expression differences across samples into a higher order network structure, elucidating relationships among genes based on their co-expression profiles [5,6].

Here, we propose to utilize a set of statistics implemented in WGCNA, called preservation statistics, to elucidate global differences in mechanisms underlying the early phase of DMD [7]. Traditionally, these statistics have been utilized to identify modules of genes that are topologically preserved between two networks. In contrast to this approach, we propose to utilize these statistics to identify modules that do not exhibit a preservation of topology between networks. This is based on the premise that such modules would represent a cohort of gene interactions that are vastly different between conditions and point to dysfunctional pathways and interactions.

In our current study we utilize a previously published dataset on DMD containing a cohort of healthy and affected individuals (mostly children) - representing the early phase of DMD development [8]. Briefly, we evaluated differential mechanisms between dystrophic and healthy skeletal muscle using the following approach; first, co-expression networks were generated independently for healthy and dystrophic samples; second, clustering each of the co-expression networks resulted in several groups of biologically relevant genes (modules) for each condition; and finally, preservation of modular topology from one condition was detected with respect to the second condition, allowing us to identify differences in gene connectivity patterns between conditions.

The results of our differential analysis reveal convergent molecular mechanisms consistent with published studies in addition to providing us novel hypothesis on gene interactions associated with the early phase of DMD.

## Results and discussion

### Network construction and modularity detection

WGCNA was utilized to construct unsigned weighted co-expression networks from 13 healthy and 23 DMD muscle samples across 4000 most varying genes (see Methods). Briefly, unsigned network adjacency matrices were obtained by raising the Pearson correlation matrices to a power β =5 for each condition [5]. The adjacencies were transformed to similarity matrices for subsequent clustering. When represented as networks, each entry of the similarity matrix *ij* corresponds to weight on the edge between genes *ij*. The strength of similarity between two genes depends not only on the correlation but also on their shared network neighborhood [5]. Clustering based on such a similarity allowed for identification of gene groups that were biologically relevant.

Hierarchical clustering of the two weighted networks resulted in eleven modules for the network from healthy samples (N1-N11, see Methods) and ten modules from the network derived from dystrophic samples (D1-D10, see Methods). Additional file 1 shows the clustering dendrograms and corresponding modules identified in both networks. Genes that did not cluster were excluded from further analysis for the purposes of this study.

### Functional characterization of modules identified in healthy and dystrophic networks

Modules identified using WGCNA have been repeatedly shown to be biological relevant to the condition under study [4,9]. We utilized functional enrichment analysis as a method to assess the functional coherence of modules identified within each of the networks.

#### Characterizing modules of the healthy network

Enrichment of modules from the healthy network revealed several functions routinely associated with healthy skeletal muscle such as striated muscle contraction, energy generation and extracellular matrix organization (Table 1).

**Table 1:** **Enrichment of modules identified in the healthy network**

**Module**	**#Nodes**	**Top Term**	**p value**
**N1**	590	striated muscle contraction	7.59E-07
**N2**	323	extracellular structure organization	2.90E-08
**N3**	109	actin cytoskeleton organization	1.50E-02
**N4**	598	modification-dependent macromolecule catabolic process	2.36E-04
**N5**	349	intracellular protein transport	7.69E-06
**N6**	93	generation of precursor metabolites and energy	8.27E-39
**N7**	215	chromatin assembly or disassembly	8.06E-04
**N8**	125	muscle organ development	3.74E-03
**N9**	171	fatty acid metabolic process	3.85E-05
**N10**	1102	intracellular protein transport	5.12E-05
**N11**	319	ribosomal small subunit biogenesis	4.28E-05

This table represents the top functional enrichment term from the highest ranking annotation cluster identified for each module of the healthy network. The annotation clusters were ranked and identified using DAVID’s annotation clustering tool [31] [see Additional file 6].

Skeletal muscle contraction occurs via the coordinated movement of several proteins particularly the actin-myosin complex within the sarcomere, incident upon a changing Ca^2+^ flux. Several genes encoding the sarcomeric proteins such as MYH2, MYH6, MYH7, MYBPC2 TPM1, TPM3, TNNC1/2, TNNI1, TNNT1, MYOM2, MYOZ1, MYOZ2, and MYOZ3 were identified in modules N1 and N8 [10]. The extracellular matrix (ECM) surrounding the skeletal muscle plays an important role in force transmission and affects the mechanical properties of the skeletal muscle. Several genes associated with the ECM and focal adhesion such as COL4A1, COL4A2, COL5A2, COL6A1, COL6A2, ITGA6, ITGB1, CAV1, CTNNB1, ACTB and LAMN4 were identified in modules N1 and N8 [11]. Muscle contraction and relaxation depend primarily upon energy derived from hydrolysis of adenosine triphosphate (ATP) within the mitochondria. Glycogen/glucose and lipid metabolism serve as major sources of ATP within muscle. Several genes associated with such metabolism were identified within modules N6 and N9 with genes such as NDUFB3, NDUFB5, FABP4, AACS, ADIPOQ, SDHA, SDHB and SCD.

#### Characterizing modules of the dystrophic network

Though the same 4000 genes were used to construct the co-expression network in each case, modules cluster differently based on their co-expression. Subsequent enrichment of modules from the dystrophic network revealed functions particularly associated with dystrophic muscle such as response to wounding (Table 2).

**Table 2:** **Enrichment of modules identified in the dystrophic network**

**Module**	**#Nodes**	**Top Term**	**p-value**
**D1**	247	cytoskeleton organization	1.89E-04
**D2**	156	response to wounding	5.09E-07
**D3**	121	blood vessel development	5.07E-03
**D4**	377	modification-dependent macromolecule catabolic process	1.56E-03
**D5**	540	ubiquitin-dependent protein catabolic process	5.24E-04
**D6**	874	generation of precursor metabolites and energy	7.03E-30
**D7**	180	muscle system process	2.67E-04
**D8**	301	RNA splicing	3.52E-10
**D9**	75	extracellular matrix organization	1.22E-05
**D10**	1089	positive regulation of ligase activity	9.73E-05

This table represents the top functional enrichment from the highest ranking annotation cluster identified for each module of the diseased network. The annotation clusters were ranked and identified using DAVID’s annotation clustering tool [31] [see Additional file 7].

For instance, module D2 contained several genes associated with wounding and inflammatory response, including several cathepsins and MHC class II antigen processing and presentation genes such as HLA-DPA1, HLA-DMA, HLA-DPB1, HLA-DQA1, HLA-DRA, and HLA-DRB1 [12]. Chronic inflammatory processes are known to initiate fibrosis within dystrophic muscle [13]. Concurrently, ECM adapts dramatically altering both the manifestation and function within dystrophic muscle. We observe the co-expression of ECM markers affecting fibrosis such as fibronectin (FN1) (a fibroblast marker) and lumican (LUM) - both known to influence collagen expression within this module [14].

Modules D4 and D5 were associated with apoptosis and proteolytic processes within the muscle - more specifically ubiquitin-proteosome system [15] with genes such as genes of the 23 s proteasome (PSMA2/3, PSMD9/12, PSME2/4), ubiquitin conjugating enzymes (UBE2B, UBE2D1), ubiquitin ligases (UBE3A, UBE3C), ubiquitin peptidases (USP11, USP6) co-expressed with cullins (CUL4A and CUL5) that serve as scaffolds for ubiquitin ligases.

### Identifying functional differences between healthy and dystrophic muscle- a systems approach

The functional annotation clustering results above suggested a mutual exclusivity of certain functions between dystrophic and healthy muscle implying a difference in the topology of connections for genes within these networks.

In order to systematically assess and quantify differential gene co-expression, we performed a “preservation” analysis. This allowed us to identify modules that were fairly unique in terms of their gene co-expression within a given network compared to another. We utilized a method implemented in WGCNA called “*modulePreservation*” [7]. In contrast to the idea of the original paper which aimed at identifying modules preserved between conditions, we aimed to identify modules “weakly preserved” across conditions (see Methods). We hypothesized that modules that were either weakly preserved or non-preserved in either condition might point to dysregulated pathways in disease that were either acquired or lost with respect to a healthy skeletal muscle.

#### Assessing differential co-expression in healthy muscle with respect to dystrophy

In order to evaluate how the topology of the healthy network differed from the dystrophic network, we computed the preservation (density, connectivity and separability statistics) of modules from the healthy network (reference network) as compared to the dystrophic network (test network). Lower preservation statistics suggested a loss of co-expression structure between these gene pairs in the dystrophic network.

**Figure 1:**
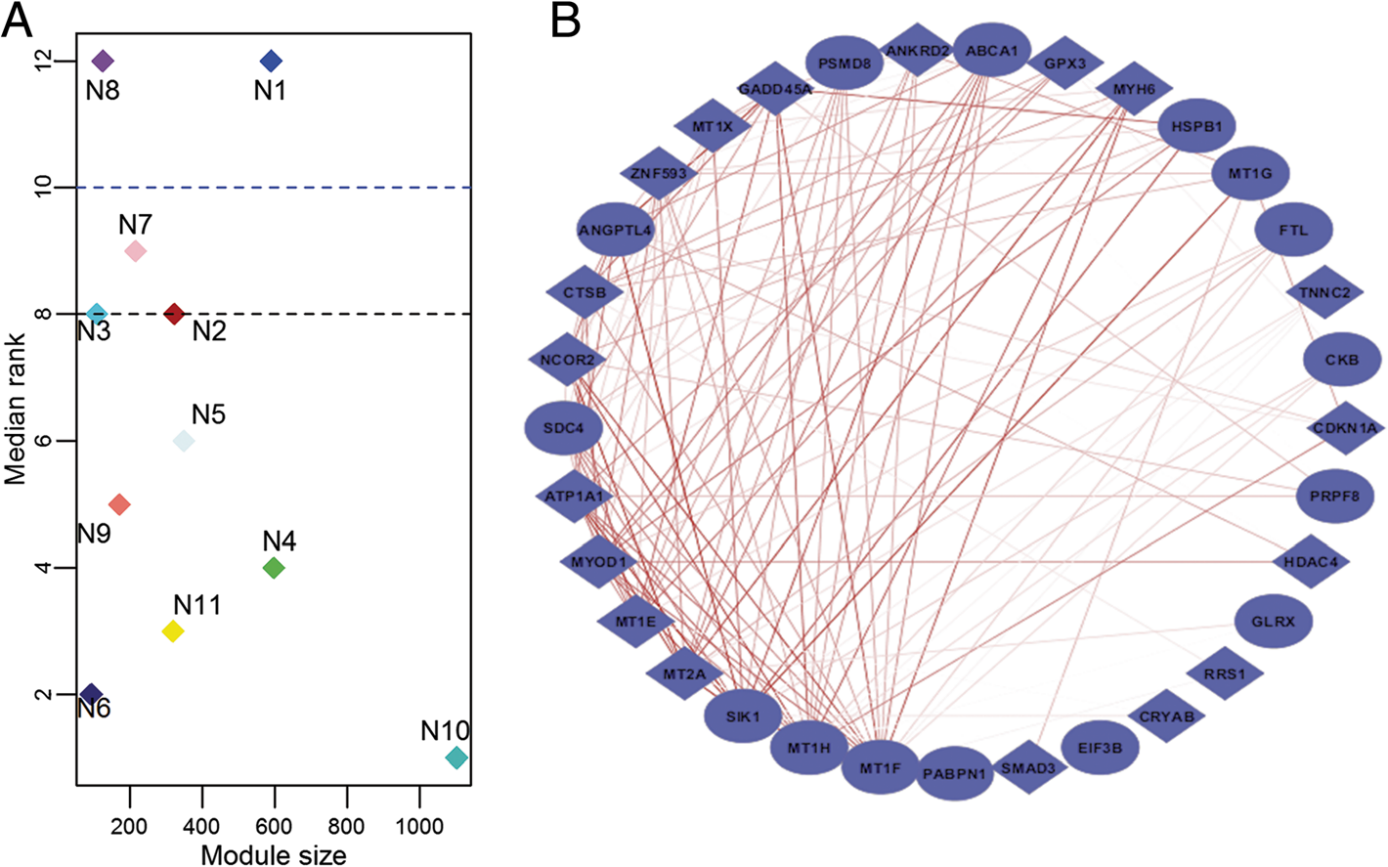
Differential co-expression in healthy muscle with respect to dystrophy. **A:** Scatter plot identifying the median rank of module preservation between test (dystrophic) and reference (healthy) networks. **B:** Co-expression between genes identified in module N1- The co-expression patterns for a subset of genes identified within module N1 are shown here. All interactions have a specificity of >0.95. Darker the line, stronger is the strength of co-expression between the gene pairs.

Based on the median preservation score, we identified a total of 5 interesting modules. Two modules N1 and N8 from the healthy network were non-preserved in the dystrophic network while three other modules N2, N3 and particularly N7 exhibited weak preservation (Figure 1A). A table of the observed preservation statistics for all modules of the healthy network is provided in Additional file 2. Z_summary_, a permutation statistic (see Methods) defined for assessing significance of the observed preservation also revealed a low preservation of these modules (Table 3). Broadly, loss of healthy muscle function and weakened contractility in dystrophic muscle triggers the activation of atrophic pathways leading to severe muscle wasting, changes to the extracellular matrix, fibrosis and necrosis over time [16]. Accordingly, unpreserved modules N1 and N8 were associated with genes necessary for striated muscle contraction, while the weakly preserved modules (N2, N3 and N7) were associated with ECM and cytoskeletal framework of the skeletal muscle.

**Table 3:** **Permutation based Z**_**summary**_

**Module**	**Size**	**Median rank**	**Z summary**	**log p values (Z summary)**
**N1**	**590**	**12**	**6.03**	**−13.80**
**N2**	**323**	**8**	**9.66**	**−29.99**
**N3**	**109**	**8**	**5.25**	**−8.66**
**N4**	598	4	23.84	−139.55
**N5**	349	6	15.97	−71.44
**N6**	93	2	10.69	−27.93
**N7**	**215**	**9**	**5.53**	**−9.57**
**N8**	**125**	**12**	**2.33**	**−2.69**
**N9**	171	5	10.26	−28.77
**N10**	1102	1	51.15	−581.53
**N11**	319	3	18.85	−91.87

This table reports the composite measure Z_summary_ and its associated p-value obtained by permuting modules labels in the dystrophic (test) network to assess preservation of modules in the healthy network. Median rank based on the observed statistics are also reported here. Data for modules identified as being weakly preserved are shown in bold.

We utilized a co-expression specificity measure [17] (see Methods) to elucidate co-expressed genes pairs (edges) from these 5 modules. We observed that modules exhibiting none-to-low preservation in the healthy network consistently had a higher fraction of gene pairs exclusive to the healthy network than their preserved counterparts (Table 4). For instance, ~35% of the gene pairs considered (599/1738) within N1 were specific to the healthy network (Figure 1B). Several of the genes involved are known markers influencing skeletal muscle contraction such as ANKRD2, TNNC2, SMAD3, HSPB1, CRYAB, SDC4, and MYOD1.

**Table 4:** **Healthy network specificity- this table represents the fraction of edges identified as being exclusive to the healthy modules as compared to the dystrophic network**

**Module Name**	**#Genes (n)**	**#Gene pairs considered**	**Gene pair specificity (%)**
**N1**	590	1738	34.46
**N2**	323	520	15.00
**N3**	109	59	16.95
**N4**	598	1785	3.08
**N5**	349	607	2.31
**N6**	93	43	6.98
**N7**	215	230	16.09
**N8**	125	78	16.67
**N9**	171	145	4.83
**N10**	1102	6067	0.12
**N11**	319	507	2.37

#Gene pairs represent the top 1% of the edges calculated as 0.01*(n(n-1)/2).

It was interesting to observe however that a majority of the genes identified as being part of these interactions were ion- binding as per GO’s molecular function ontology (zinc and copper, p < 10^−4^). A visual inspection of subset of the exclusive genes pairs identified reveals strong co-expression between several zinc binding genes such as metallothioneins (MT1E/F/H/X), ZNF593, and genes affecting muscle contraction (ANKRD1, MYOD1, SMAD3, HSPB1). Metallothioneins have been postulated to be associated with a host of functions ranging from chaperones for synthesis of metalloproteins, to reservoirs of essential metals (Zn and Cu) in healthy tissue [18]. Specifically, metallothioneins (MTs) exhibit specific redox properties and have been speculated to selectively control release and uptake of Zinc [18]. However, it is interesting to note that MTs were co-expressed with genes affecting muscle contraction only within the healthy network, suggesting a link between zinc homeostasis, and muscle contraction in healthy muscle. The exclusivity of connections to the healthy network further emphasizes the possibility of an aberration in zinc homeostasis and its effect on contraction in DMD.

#### Assessing differential co-expression in dystrophic muscle with respect to healthy tissue

**Figure 2:**
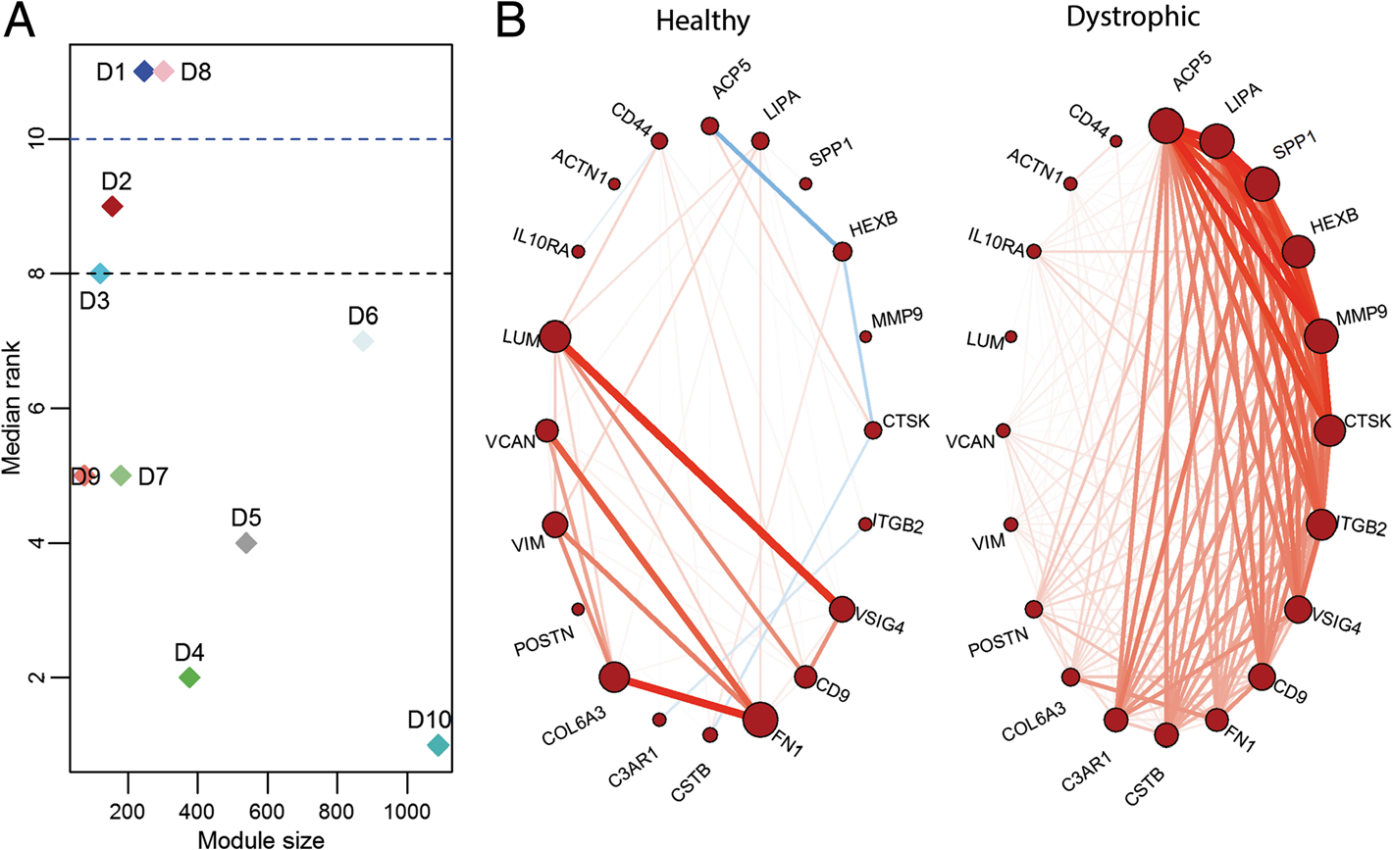
Differential co-expression in dystrophic muscle with respect to healthy muscle. **A:** Scatter plot identifying the median rank of module preservation between test (healthy) and reference (dystrophic) networks. **B:** The Pearson correlation between a subset of genes identified in module D2 from the dystrophic network- For the same set of genes from module D2, we also identify correlation patterns in the healthy network (left). The size of the node is proportional to the sum of all correlation strengths at the node in the network shown. Red lines indicate positive correlation and blue indicate negative correlation.

A similar analysis with dystrophic network as the reference network, allowed us to identify gene pairs that were not conserved in healthy tissue. As proposed earlier, we speculated that identifying non/weakly preserved modules in the dystrophic network could point to gene associations that are *gained* in dystrophy. We identified two modules- D1 and D8 that exhibited no preservation in the healthy network while two other modules D3 and to a greater extent D2 were weakly preserved (Figure 2A). A table of the observed preservation statistics for all modules of the dystrophic network is provided in Additional file 3. The Z_summary_ scores (see Methods) likewise revealed a low preservation of these modules via permutation testing (Table 5).

**Table 5:** **Permutation based Z**_**summary**_

**Module**	**Size**	**Median rank**	**Z summary**	**log p values (Z summary)**
**D1**	**247**	**11**	**2.50**	**−3.03**
**D2**	**156**	**9**	**2.35**	**−2.78**
**D3**	**121**	**8**	**7.36**	**−17.83**
**D4**	377	2	16.89	−73.04
**D5**	540	4	20.28	−113.58
**D6**	874	7	18.91	−120.35
**D7**	180	5	9.60	−28.01
**D8**	**301**	**11**	**5.36**	**−12.79**
**D9**	75	5	4.80	−6.16
**D10**	1089	1	50.95	−568.57

This table reports the composite measure Z_summary_ and its associated p-value obtained by permuting modules labels in the healthy (test) network to assess preservation of modules in the dystrophic network. Median rank based on the observed statistics are also reported here. Data for modules identified as being weakly preserved are shown in bold.

Juvenile dystrophic muscle, in general, exhibits atrophy and is pre-necrotic, with pathways associated with wounding and inflammation being subsequently activated. The functional enrichment identified within these four modules (Table 2) corroborated our approach, highlighting functions that are more pronounced in dystrophic muscle compared to healthy tissue.

These modules also exhibited higher specificity of connections to the dystrophic network than their preserved counterparts (Table 6). For instance, the highest specificity was observed for module D2 with nearly 45% of its gene pairs as being specific to dystrophy (specificity > 0.95). Interestingly, the dystrophic-specific gene interactions identified in module D2 corresponded with interactions categorized as a part of the inflammatory and tissue repair/remodeling repertoire of genes, as witnessed in models of skeletal muscle injury, particularly dystrophy (Figure 2B).

**Table 6:** **This table lists the fraction of edges identified as being exclusive to the dystrophic modules with respect to the healthy network**

**Module Name**	**#Genes**	**#Gene pairs**	**Specificity (%)**
**D1**	247	304	20.39
**D2**	156	121	45.45
**D3**	121	73	4.11
**D4**	377	709	2.12
**D5**	540	1455	4.60
**D6**	874	3815	3.96
**D7**	180	161	3.11
**D8**	301	452	3.32
**D9**	75	28	0.00
**D10**	1089	5924	0.20

#Gene pairs represent the top 1% of the edges calculated as 0.01*(n(n-1)/2).

 For instance, expression of SPP1, a multifunctional cytokine (also called early T-cell activation-1 (Eta-1), osteopontin), is linked with macrophage infiltration, resulting in a chronic inflammatory response observed in dystrophic muscle [13,19]. VSIG4, a regulator of T-cell activation expressed mostly in macrophages is strongly co-expressed within D2 [19]. Though the exact mechanisms by which skeletal muscle attracts and allows entry of neutrophils and macrophages in dystrophic muscle are not well understood, there is evidence suggesting that ITGB2 is required to control the functional activities of neutrophils and macrophages within muscle [20]. Fibrosis observed in DMD, is largely activated in response to chronic inflammatory processes initiated within dystrophic muscle [13] and broadly refers to the accumulation of excess connective tissue (ECM) [11]. SPP1 which is also expressed in fibrotic lesions is considered a marker for disease severity in DMD [21]. SPP1 is required for differentiation of myofibroblasts [22], an important class of fibroblastic cells required for wound healing, present abundantly within dystrophic muscle. Fibronectin (FN1) serves as a marker for fibroblast activation in muscle [23].

SPP1, in addition to modulating fibrotic responses, promotes cell-cell and cell-matrix adhesions through its interaction with integrins, and CD44 [24]. Mature focal adhesion complexes containing genes such as ACTN1, fail to form in the absence of SPP1 (SPP1^−/−^) within myofibroblast cultures [22] suggesting similar pathways for adhesion in dystrophy. Interestingly, within this module we also identify MMP9- a matrix metalloproteinase whose increased expression, particularly in the pathology of DMD, is associated with breakdown of cytoskeleton-ECM components leading to sarcolemmal damage and fiber necrosis [25,26] Additionally, MMP9 is also suggested to act as an inflammatory stimulus for mediating neutrophil and macrophage infiltration within the dystrophic skeletal muscle [27,28].

SPP1 is subject to extensive posttranslational modification via glycosylation, phosphorylation and sulphation. Specific posttranslational modifications have been associated with altered properties and function of SPP1 [29]. Interestingly, ACP5, a phosphatase required for mineralization of cartilage and bone matrix resorption [28] was recently demonstrated to be responsible for phosphorylation of SPP1 in endometrial tissue. Though no evidence for role of ACP5 or post-translation modification of SPP1 in dystrophic muscle exists, the co-expression of ACP5 with SPP1 suggests a possible role in dystrophy warranting further investigation.

Additionally, SPP1 shows high specificity interactions with certain ECM markers including CTSK, LUM, VCAN and VIM (Figure 2B). Though there is no direct evidence for the interaction of these markers with SPP1, the extant understanding of the ECM markers combined with the high specificity of co-expression observed in our network module suggest possible associations with SPP1 in dystrophic muscle.

Overall, our results indicate that the modules exhibiting low preservation statistics contain several gene pairs that are likely to be associated with the disease progression. Though it is conceivable that not all the genes identified within these less-preserved modules play a role in disease, several high specificity gene pairs identified were noted and hypothesized to play a significant role in pathogenesis of DMD.

## Conclusions

An analysis of modules exhibiting a low preservation between dystrophic and healthy conditions showed that these modules showed a higher specificity among gene pairs pertinent to the condition under study. We illustrated the application of using preservation statistics to detecting modules functionally associated with dysregulated pathways in disease, as exemplified by the inflammatory module D2. This approach enabled identifying putative biomarkers, such as ACP5 identified within module D2, likely to be associated with the disease.

In summary, our method provided a simple approach to identifying differences between conditions, which can be utilized for exploratory analysis of dysregulated pathways in disease using a published set of statistics.

## Methods

### Data acquisition

The raw (.CEL) files for GSE6011 was downloaded from GEO [30]. This data consists of 37 Affymetrix HG-U133A microarrays with 24 juvenile DMD samples, between ages 1.5-61 months, and 13 age matched controls [8].

### Data processing

The data set was preprocessed using Bioconductor/R packages *affy* and *WGCNA*. Data was MAS 5.0 normalized using functions in *affy* and any array with an average inter sample correlation <2 SDs (σ) below the mean was removed [9]. This resulted in the removal of a single array - GSM139506.CEL (2.54 σ below mean) from the study. All probes with missing Entrez gene identifiers were excluded from this study, resulting in a data set comprising of the expression values for 11101 probes. Multiple probes were collapsed into a gene based on variance resulting in a final reduced expression data set comprising of 7996 genes and 36 samples. A subset of 4000 most varying genes was used to construct the co-expression networks, in an effort to minimize computational complexity and eliminate low varying genes that may contribute minimally to the co-expression matrix. The number 4000 was chosen as it represents roughly half the total number of genes (7996 genes) identified after pre-processing. This method of gene list selection is agnostic to their pathophysiological role in the muscle.

### Co-expression network generation and modularity detection

The topological overlap measure (TOM) in WGCNA between genes i and j is defined as follows

$$ TO{M}_{ij}=\frac{{\displaystyle {\sum}_{k=1}^N}{A}_{i,k}.{A}_{k,j}+{A}_{i,j}}{ \min \left({k}_{i,}{k}_j\right)+1-{A}_{i,j}} $$

Where A is the weighted adjacency matrix given by *A*_*ij*_ = |*cor*(*x*_*i*,_*x*_*j*)_|^*β*^ and β ≥ 1 is the soft thresholding power.

TOM takes continuous values between 0 and 1, where 0 for a gene pair indicates no similarity between the genes while 1 indicates a direct link. The soft thresholding power β for each dataset in our study was ascertained as prescribed in the original publication [5].

Co-expression networks from the adjacency matrices of healthy and dystrophic samples were generated using the “TOMsimilarity” function available via WGCNA. Hierarchical clustering on the topological dissimilarity (1-TOM) was performed using the function “flashClust”. The tree cut height was dynamically determined using the function “cutreeDynamic” in WGCNA, for identifying modules in each of our networks. Additional files 4 and 5 provide a list of all genes identified in each of the networks (healthy and dystrophic respectively) and their corresponding module assignments.

### Preservation of modules

“Module preservation” or preservation statistics implemented in WGCNA allows us to detect the conservation of gene pairs between two networks (test and reference) [7]. Briefly, three types of network based module preservation statistics have been identified by this method, namely

1. Density based preservation statistics: determine if nodes remain highly connected in the test network. Four independent measures account for this statistic.
2. Connectivity based preservation statistics determine the extent to which connectivity patterns between nodes in the reference network are similar to the test network. Three independent measures of the network account for this statistic.
3. Separability based preservation statistics determine if network modules remain distinct from one another in the test network.

Network based statistics employed by WGCNA do not require identification of modules within the test network to ascertain the conservation of reference network modules within the test network. This is in contrast to several existing methods that ascertain module preservation as discussed in the original publication. The authors of the original publication have shown that using this method it is possible to identify sets of preserved co-expression across species.

As these statistics measure distinct aspects of module preservation, two composite measures have been defined

1. Median rank: A composite measure that is based on observed preservation values and is less dependent on module sizes. It is defined as the mean of median ranks computed for connectivity and density measures of each module (0.5 (medianRank_connectivity_ + medianRank_density_).
2. Z_summary_: A permutation based composite Z statistic that is used to assess the significance of observed statistics and is defined as the mean of Z scores computed for density and connectivity measures (0.5(Z_denstiy_ + Z_connectivity_)). An associated empirical p-value is also calculated by the algorithm.

We utilize median rank to identify module preservation and Z_summary_ to assess significance of module preservation via permutation testing. Based on the number of modules within each of our networks, a median rank of 8 was chosen as a cutoff to detect weak preservation. Permutation was performed 200 times given the computational complexity involved for our network sizes. Based on the thresholds prescribed in [7], modules with a Z_summary_ score >10 indicate preservation, 2 to 10 indicate weak to moderate preservation and <2 indicate no preservation in the permutations.

### Network specific gene pairs

Condition specific interactions for a given pair of genes *i* and *j* was defined as [17]:

$$ Specificit{y}_{cond1}=\frac{TO{M}_{ij(cond1)}}{TO{M}_{ij(cond1)}+TO{M}_{ij(cond2)}} $$

Where, TOM_ij (cond)._ is the normalized TO for the gene pairs *i-j* in the given condition (healthy or disease).

We considered gene pairs to be condition specific, if the specificity was >0.95 and were in the top 1% of the gene pairs ranked on TOM similarity in any given module. Number of edges in an undirected network is computed as n(n-1)/2, where n is the number of nodes. Considering the top 1% allowed us to focus only on the strongest co-expression patterns within the module, rather than noise.

### Enrichment analysis and visualization

The results presented correspond to the top term identified in the highest-ranking cluster (as of this analysis) using the annotation clustering feature available in DAVID [31], with Gene Ontology’s Biological process functional annotations. Additional files 6 and 7 provide the top 3 functional annotation clusters identified for each of the modules within the healthy and dystrophic networks respectively. Cytoscape [32] and Bioconductor [33] were utilized for generating the figures in this paper.

### Availability of supporting data

The data sets supporting the results of this article are published data sets available through the Gene expression omnibus repository, [GSE6011 http://www.ncbi.nlm.nih.gov/geo/query/acc.cgi?acc=GSE6011].

## Additional files

Additional file 1:
**Hierarchical clustering results for the a) Healthy (normal) Network and b) Dystrophic (DMD) network: The upper section represents the cluster dendrogram of the differentially expressed genes identified for a) and b).** The lower section (bar charts) indicates the modules identified and their respective sizes after hierarchical clustering. Each module is represented by the same colors in the dendrogram for ease of visualization. Additional file 2:
**This file shows the observed preservation statistics computed using “modulePreservation” in WGCNA: The preservation of the modules of the healthy network (NRML- reference network) are identified in the entire dystrophic network (DMD- test network).**
Additional file 3:
**This file shows the observed preservation statistics computed using “modulePreservation” in WGCNA: The preservation of the modules of the dystrophic network (DMD- reference network) are identified in the entire healthy network (NRML- test network).**
Additional file 4:
**This .csv file provides the list of all genes, their entrez ids and a corresponding module assignment vector.** Module names as colors, identifiable by WGCNA are also provided for the healthy network. Additional file 5:
**This .csv file provides the list of genes, their entrez ids and a corresponding module assignment vector.** Module names as colors, identifiable by WGCNA are also provided for the dystrophic network. Additional file 6:
**This file provides the top 3 functional annotation clusters identified using DAVID, for each module of the healthy network.**
Additional file 7:
**This file provides the top 3 functional annotation clusters identified using DAVID, for each module of the dystrophic network.**

## Abbreviations

DMD: Duchenne muscular dystrophy
ECM: Extracellular Matrix
GEO: Gene expression omnibus
MT: Metallothioneins
WGCNA: Weighted gene co-expression network analysis
