# Supplementary material for: Dysregulated mechanisms underlying Duchenne muscular dystrophy from co-expression network preservation analysis
Source: BMC Res Notes. 2015 May 3;8:182. doi: 10.1186/s13104-015-1141-9 (PMC4424514; doi:10.1186/s13104-015-1141-9)
Supplement: Additional file 1: — Hierarchical clustering results for the a) Healthy (normal) Network and b) Dystrophic (DMD) network: The upper section represents the cluster dendrogram of the differentially expressed genes identified for a) and b). The lower section (bar charts) indicates the modules identified and their respective sizes after hierarchical clustering. Each module is represented by the same colors in the dendrogram for ease of visualization. [file 13104_2015_1141_MOESM1_ESM.docx]

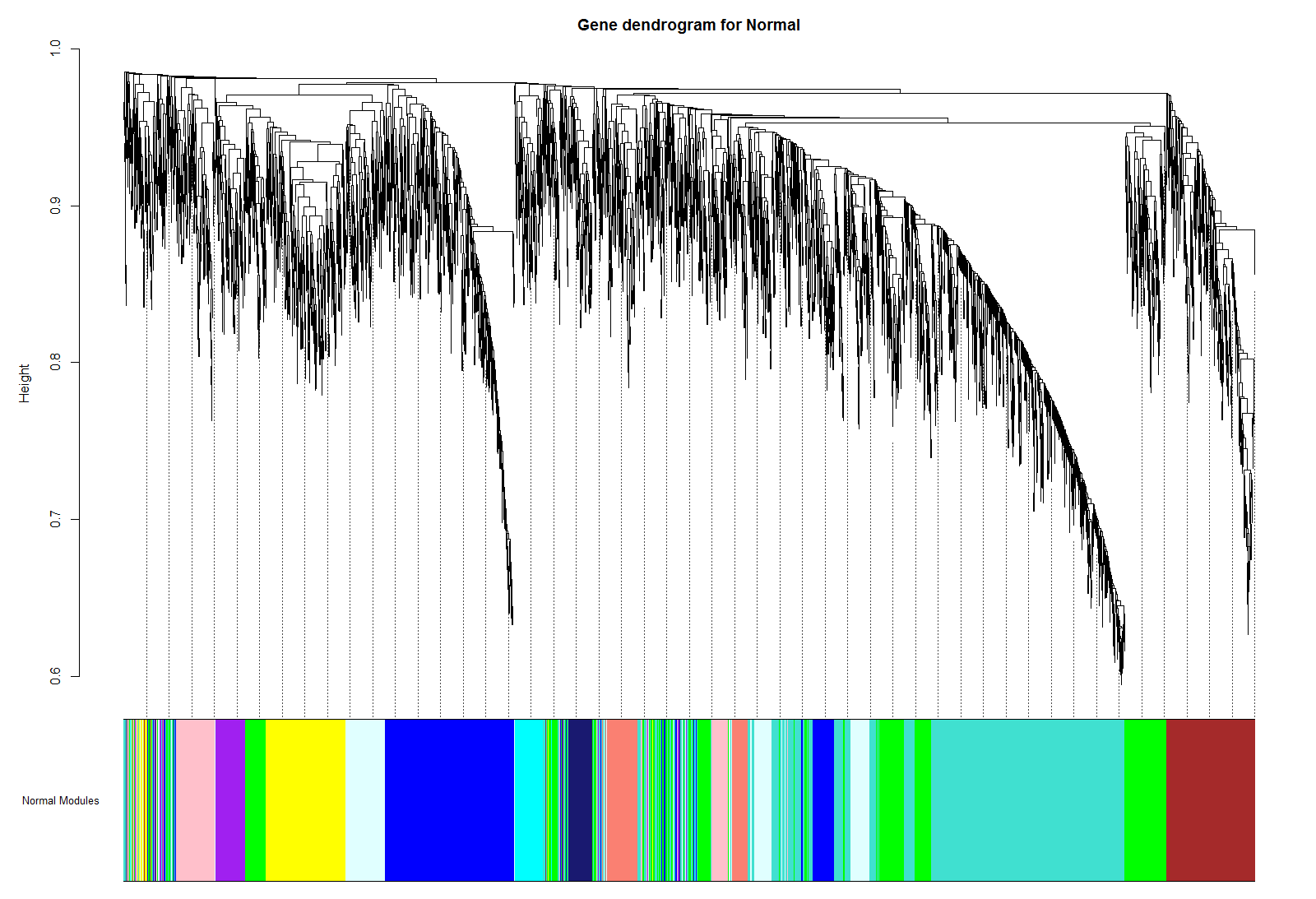

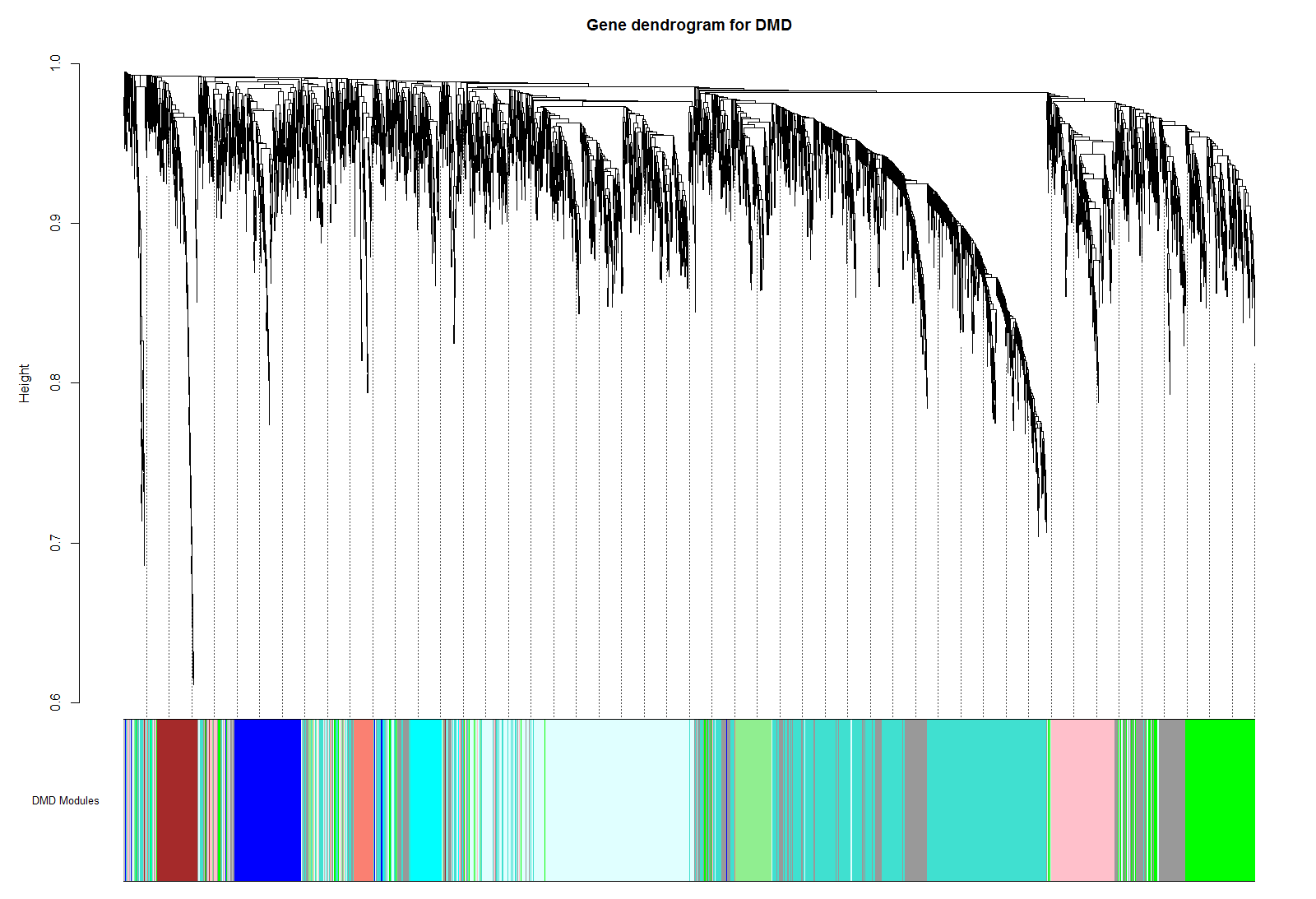


Additional file 1- Hierarchical clustering results for the a) Healthy (normal) Network and b) Dystrophic (DMD) network: The upper section represents the cluster dendrogram of the differentially expressed genes identified for a) and b). The lower section (bar charts) indicates the modules identified and their respective sizes after hierarchical clustering. Each module is represented by the same colors in the dendrogram for ease of visualization.
